# A New Multiple Hypothesis Tracker Using Validation Gate with Motion Direction Constraint

**DOI:** 10.3390/s20174816

**Published:** 2020-08-26

**Authors:** Jinping Sun, Ziwei Wang, Qing Li

**Affiliations:** 1 School of Electronics & Information Engineering, Beihang University, Beijing 100191, China; sunjinping@buaa.edu.cn (J.S.); wangziwei@buaa.edu.cn (Z.W.); 2 Department of Engineering, University of Cambridge, Cambridge CB2 1PZ, UK

**Keywords:** validation gate, multiple hypothesis tracker, motion direction constraint, clutter density estimator

## Abstract

In multi-target tracking scenarios with dense and heterogeneous clutter, there is a substantial increase in the false measurements that originated from the clutter within the validation gate, and consequently, the number of measurement-to-track association hypothesis grows rapidly in traditional multiple hypothesis tracker (MHT), leading to a sharp decrease in data association accuracy and tracking performance. A new multiple hypothesis tracker using validation gate with motion direction constraint (MHT-MDC) is proposed to solve these problems. In the MHT-MDC, a motion direction constraint (MDC) gate is designed by considering the prior target maneuvering information, which effectively reduces the volume of validation gate and, thus, diminishes the number of false measurements in the gate when the innovation covariance is large. Subsequently, the clutter density in the MDC gate is adaptively estimated by the conditional mean estimator of clutter density (CMECD), based on which the score functions in the MDC gate can be calculated. The MHT-MDC is compared with the MHT algorithm in simulations, and the experimental results demonstrate its superior tracking performance for weakly maneuvering targets in high clutter density scenarios.

## 1. Introduction

Multi-target tracking in clutter scenes is of great significance in the field of radar data processing [1,2]. The existence of clutter increases the uncertainty of measurement source; therefore, a data association algorithm is required in order to determine whether the measurement is the clutter and, if not, further specify from which target it originates. Multiple hypothesis tracker (MHT) is a Bayesian data association algorithm with a deferred decision logic [3,4,5], which theoretically provides an optimal solution to data association. The benefits of utilizing MHT is that not only can it detect the target birth/death, but it also achieves better multi-target tracking performance in complex scenes with dense clutter and targets. The MHT algorithm is normally categorized into two types: the hypothesis-oriented MHT (HOMHT) [3,4] and the track-oriented MHT (TOMHT) [5], in which the TOMHT algorithm has been widely employed due to its simplicity in trajectory generation and computation efficiency.

Despite that the TOMHT algorithm can tackle the data association problem approximately optimally, it involves an intractable growth of permutations for possible measurement-to-track association hypotheses as time evolves, which makes the data association calculations infeasible. Validation gate techniques are adopted in order to eliminate unlikely association hypotheses to reduce the computation of data association. Particularly, the center of the validation gate lies in the one-step predicted position, with its gate size mainly defined by parameters, such as the existence probability of target and the innovation covariance. The validated measurements are measurements fallen in the validation gate, and the gate size determines the number of validated measurements. If the gate size is too small, the probability of missing the target-originated measurement will increase, which will decrease the accuracy of data association; however, if it is too big, a large number of non-target-measurements will fall in the validation gate, which will lead to a large computational load and poor tracking performance.

With the increasing complexity of the radar observation scenarios, the contradiction between increasing the existence probability of target in the gate and diminishing the clutter number within the gate becomes more significant when using traditional validation gate methods. Such conflict leads to the degeneration of data association accuracy and tracking performance. The design of the validation gate has been continually improved to enhance the accuracy of data association in the clutter scenarios in order to solve these problems. In [6,7,8], the authors presented the adaptive optimal or local optimal validation gate size estimation algorithms. An improved sequential Monte Carlo (SMC) implementation of the probability hypothesis density (PHD) filter was proposed in [9], and a sigma-gate was constructed to eliminate the contribution of measurements locating outside the gate around the particle, which makes it obtain much faster processing speed and higher estimation accuracy than the standard PHD filter. An improved probability data association (PDA) algorithm was proposed in [10], which first sorts validated measurements according to the measurement likelihoods and then selects the first *K* measurements for the PDA processing. In literature [11], a fast and non-elliptical validation gate based on target maneuvering information was proposed for an application in a marine ship monitoring system with a low data rate. This method effectively improves the existence probability of maneuvering targets within the validation gate; however, it increases the computation complexity of data association. Based on the traditional elliptical gate, a circular gate using the estimated target velocity was designed in [12], in which the validated measurements are further selected by the velocity threshold and then provided to the PDA algorithm. The above validation gates enhance the target tracking performance in homogeneous clutter scenes; however, they are mainly specified for the PDA algorithm, and their multi-target tracking performance has not been verified in dense clutter cases. Besides, the heterogeneous clutter scenarios are not taken into consideration in these methods.

A new multiple hypothesis tracker using validation gate with motion direction constraint (multiple hypothesis tracker using validation gate with motion direction constraint (MHT-MDC)) is proposed under the TOMHT framework in order to reduce the number of false alarm tracks thereby enhancing the accuracy of data association in dense and heterogeneous clutter scenarios. On the basis of the traditional ellipsoidal gate, the MHT-MDC applies the prior target maneuvering information to construct the motion direction constraint (MDC) gate. Similar to other validation gate methods, here only the measurements selected by the MDC gate are associated with the tracks while the others are eliminated. Therefore, when the measurement innovation covariance is large, the proposed MDC gate technique can successfully reduce the false alarms within the gate due to the fact that the volume of the MDC gate is much smaller than other ellipsoidal gates. For heterogeneous clutter cases, a conditional mean estimator of clutter density (CMECD) is employed in order to improve the accuracy of data association which adaptively estimates the clutter density in the MDC gate, and calculates the score function of track hypothesis. The results show that our MHT-MDC algorithm can obtain better target tracking performance in heterogeneous and dense clutter scenarios.

The rest of this letter is organized, as follows. Section 2 introduces the validation gate with motion direction constraint method. In Section 3, we present an approximate method for calculating the volume of the MDC gate. In Section 4, the MHT-MDC algorithm is proposed, and its implementation process is introduced in detail. Especially, the adaptive clutter density and the adaptive score function are calculated for dense and heterogeneous clutter scenarios. In Section 5, the tracking performance of MHT-MDC is compared with the MHT algorithm. Finally, conclusions are drawn in Section 6.

## 2. Validation Gate with Motion Direction Constraint

At scan *k*, the motion of each target is modeled as,

(1) x(k)=Fx(k−1)+v(k),

where x(k) is the target state at time *k*, F is the system transition matrix, and v(k) is a zero-mean white Gaussian noise with covariance Q(k).

The observation model is

(2) z(k)=Hx(k)+w(k),

where z(k) denotes the target measurement at time *k*, H is the measurement matrix, and w(k) denotes a zero-mean white Gaussian noise with covariance R(k).

In most multi-target tracking systems, the target state and the measurement are expressed in a Cartesian coordinate system *X-Y-Z*. For target measurement expressed in a polar coordinate system, it can be conveniently converted to the *X-Y-Z* coordinate system. Thus, here we adopt the Cartesian coordinate system and constant velocity (CV) motion model for each target. At scan *k*, the target state is denoted as $x(k)={\left[x\;\dot{x}\;y\;\dot{y}\;z\;\dot{z}\right]}^{T}$, which contains both position and velocity in *X-Y-Z* axes. More specifically, the state transition matrix F and the measurement matrix H can be written as

$$F=\left[\begin{array}{ccc} F^{1} & 0 & 0\\ 0 & F^{1} & 0\\ 0 & 0 & F^{1} \end{array}\right],\;F^{1}=\left[\begin{array}{cc} 1 & T\\ 0 & 1 \end{array}\right],\;H=\left[\begin{array}{cccccc} 1 & 0 & 0 & 0 & 0 & 0\\ 0 & 0 & 1 & 0 & 0 & 0\\ 0 & 0 & 0 & 0 & 1 & 0 \end{array}\right].$$

The covariance matrix of the process noise Q, and the covariance matrix of measurement noise R are defined, as follows,

$$Q=\left[\begin{array}{ccc} Q^{1} & 0 & 0\\ 0 & Q^{1} & 0\\ 0 & 0 & Q^{1} \end{array}\right]\delta_{v}^{2},\;Q^{1}=\left[\begin{array}{cc} \frac{1}{4}T^{4} & \frac{1}{2}T^{3}\\ \frac{1}{2}T^{3} & T^{2} \end{array}\right],\;R=\left[\begin{array}{ccc} 1 & 0 & 0\\ 0 & 1 & 0\\ 0 & 0 & 1 \end{array}\right]\delta_{\varepsilon }^{2},$$

where T is the sample time interval, $\delta_{v}$ is the standard deviation of the process noise, and $\delta_{\varepsilon }$ is the standard deviation of the measurement noise.

By using Kalman filtering, the predicted measurement $\hat{z}(k|k-1)$ can be obtained at scan *k*. Then, the measurement innovation and its covariance can be calculated as

(3) $$v(k)=z(k)-\hat{z}(k|k-1),$$

(4) $$S(k)=HP(k|k-1)H^{T}+R(k),$$

where P(k|k−1) is the prediction covariance.

The gating technique is developed to limit the number of measurement-to-track pairings such that the data association computational load can be reduced. Generally, the validation gate is centered at the one-step predicted measurement $\hat{z}(k|k-1)$ and is described by an ellipsoid region,

(5) $$V_{G}=\left\{z(k)|d^{2}=\left({\left(z(k)-\hat{z}(k|k-1)\right)}^{T}S{\left(k\right)}^{-1}\left(z(k)-\hat{z}(k|k-1)\right)\leq G\right)\right\}.$$

where G is a validation gate threshold. Only the measurements that satisfy Equation (5) can be associated with the track. In the *X-Y-Z* coordinate system, the existence probability of target in the gate [1] is calculated as

(6) $$P_{G}=\int_{0}^{G}\frac{1}{\sqrt{2\pi }}x^{1/2}e^{-x/2}dx.$$

For example, if the gate threshold is G=16, the probability of the target existing in the gate is $P_{G}=0.9989$.

The volume of the validation gate is defined as

(7) $$V_{G}=\frac{4\pi G^{3/2}}{3}\sqrt{\left|S(k)\right|}.$$

Consequently, if the clutter density in the validation gate is λ(k), the clutter number in the gate is computed as $\lambda (k)V_{G}$.

In general, we seek to attain a higher existence probability of target $P_{G}$ in the gate with less clutter, so as to acquire better tracking performance. Based on Equations (6) and (7), once $P_{G}$ is determined, G is accordingly fixed, and a large innovation covariance will lead to a large validation gate volume. Hence, in a clutter dense scenario where the innovation covariance is large, false alarm measurements in the validate gate will also increase, leading to the deterioration of the tracking performance.

In real-life target tracking applications, we may possess some prior target maneuvering information. For example, targets, such as civil aircraft and vessels, are known to move with a lower possibility of strong maneuvers; in other words, the motion direction of these targets will not change dramatically at the adjacent sampling time. Such prior motion direction can be utilized to select validated measurements under the criteria that only the validated measurements within a specific motion direction gate are used for association processing with others being excluded.

Assume that at scan *k*, a set of $M_{k}$ measurements $z_{m}(k),m=1,2,\cdots ,M_{k}$ are selected by the validation gate (3). Based on the target state estimation $\hat{x}(k-1)$ at scan *k* − 1, the predicted target motion direction vector can be calculated as

(8) $$d_{k}=\frac{\hat{z}(k|k-1)-H\hat{x}(k-1)}{\left\|\hat{z}(k|k-1)-H\hat{x}(k-1)\right\|}.$$

The angle between the motion direction of validated measurement $z_{m}(k)$ and $d_{k}$ can be calculated as

(9) $$\theta_{m}=\cos^{-1}\frac{\left(z_{m}(k)-H\hat{x}(k-1)\right)\cdot d_{k}}{\left\|z_{m}(k)-H\hat{x}(k-1)\right\|}.$$

The motion direction threshold $\theta_{G}$ is set according to the prior target maneuvering information, and, thus, the motion direction constraint (MDC) gate can be defined as

(10) $$V_{\theta -G}=\left\{z_{m}(k)|\theta_{m}\leq \theta_{G}\right\}\cap V_{G}.$$

Based on the Equation (10), the MDC gate is constructed as the intersection part of the traditional ellipsoid gate and a cone region with a vertex of $H\hat{x}(k-1)$ and vertex angle being $2\theta_{G}$. The volume of the MDC gate, by definition, is less than or at most equal to the volume of the ellipsoid region; especially when the motion direction threshold is small or the innovation covariance is large, it is significantly smaller than that of the ellipsoid gate. Consequently, it reduces the number of false alarm measurements effectively thereby lowering the miscorrelation ratio of true tracks and improving the multi-target tracking performance.

## 3. The Volume of the MDC Gate

In the MHT algorithm, the volume of the gate is used to calculate the estimated clutter density. However, theoretically, the volume of the MDC gate defined in Equation (10) does not have an analytic solution. Thus, this section presents an approximate method for calculating the volume of the MDC gate.

The ellipsoid equation of the correlation gate in (5) is defined as

(11) $$v{\left(k\right)}^{T}S{\left(k\right)}^{-1}v(k)=G,$$

where the center of the validation gate is $\hat{z}(k|k-1)$. Here we specifically discuss the ellipsoid characteristics in a three-dimensional space. Firstly, the eigenvalue decomposition of the innovation covariance matrix is performed as $S(k)=U^{T}(k)E(k)U(k)$, where the diagonal matrix $E(k)=\mathrm{diag}[\lambda_{1,k}\;\lambda_{2,k}\;\lambda_{3,k}]$, the eigenvalue of S(k) is $\lambda_{1,k},\lambda_{2,k},\lambda_{3,k}$, and the unitary matrix $U(k)=[u_{1}(k)\;u_{2}(k)\;u_{3}(k)]$ with the property of $U(k)=U^{-1}(k)=U^{T}(k)$ [13]. Thus, Equation (11) can be converted to

(12) $${\left(U(k)v(k)\right)}^{T}\;E{\left(k\right)}^{-1}\left(U(k)v(k)\right)=G.$$

Set $\tilde{v}(k)={\left[{\tilde{x}}_{k}\;{\tilde{y}}_{k}\;{\tilde{z}}_{k}\right]}^{T}=U(k)v(k)$ in the new $\tilde{X}\text{-}\tilde{Y}\text{-}\tilde{Z}$ coordinate system. Subsequently, Equation (12) can be expressed as a standard ellipsoid equation,

(13) $$\frac{{\tilde{x}}_{k}^{2}}{a^{2}}+\frac{{\tilde{y}}_{k}^{2}}{b^{2}}+\frac{{\tilde{z}}_{k}^{2}}{c^{2}}=1.$$

The length of the semiaxis of the ellipsoid in the directions of $\tilde{x}$, $\tilde{y}$, and $\tilde{z}$ can be represented as $a=\sqrt{\lambda_{1,k}G}$, $b=\sqrt{\lambda_{2,k}G}$, $c=\sqrt{\lambda_{3,k}G}$.

Assume that the estimation of the target state at the previous scan $\hat{x}(k-1)$ is located at point *P* in the $\tilde{X}\text{-}\tilde{Y}\text{-}\tilde{Z}$ coordinate system after the translation-rotation, and the coordinates of *P* can be expressed as

(14) $$\tilde{x}(k)={\left[{\tilde{x}}_{p,k}\;{\tilde{y}}_{p,k}\;{\tilde{z}}_{p,k}\right]}^{T}=U(k)\left(H\hat{x}(k-1)-\hat{z}(k|k-1)\right).$$

**Figure 1:**
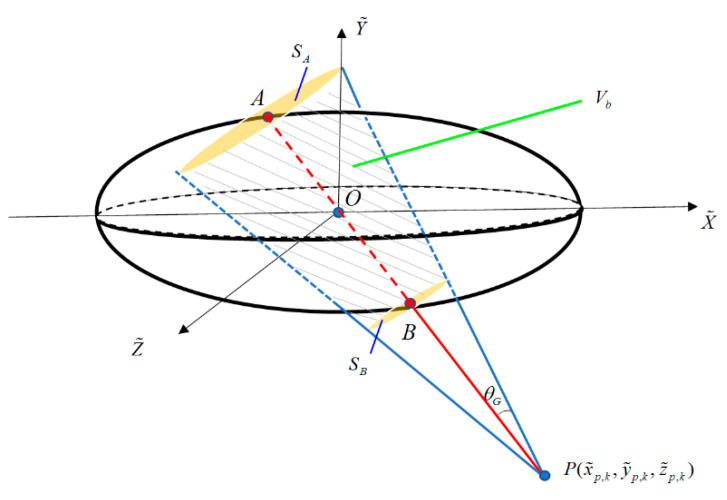
The schematic diagram of the MDC gate volume.

As shown in Figure 1, in the $\tilde{X}\text{-}\tilde{Y}\text{-}\tilde{Z}$ coordinate system, the equation of the straight line determined by point *P* and the origin O is

(15) $$\frac{{\tilde{x}}_{k}^{}}{{\tilde{x}}_{p,k}}=\frac{{\tilde{y}}_{k}^{}}{{\tilde{y}}_{p,k}}=\frac{{\tilde{z}}_{k}^{}}{{\tilde{z}}_{p,k}}.$$

By combining Equations (13) and (15), the intersection points between the line and the ellipsoid are $A({\tilde{x}}_{A},\;{\tilde{y}}_{A},\;{\tilde{z}}_{A})$, $B({\tilde{x}}_{B},\;{\tilde{y}}_{B},\;{\tilde{z}}_{B})$. If ${\tilde{x}}_{p,k}\geq 0$, then

(16) $$\{\begin{array}{l} {\tilde{x}}_{A}=-\zeta \\ {\tilde{y}}_{A}=-\left({\tilde{y}}_{p,k}/{\tilde{x}}_{p,k}\right)\zeta \\ {\tilde{z}}_{A}=-\left({\tilde{z}}_{p,k}/{\tilde{x}}_{p,k}\right)\zeta \end{array},\;\{\begin{array}{l} {\tilde{x}}_{B}=\zeta \\ {\tilde{y}}_{B}=\left({\tilde{y}}_{p,k}/{\tilde{x}}_{p,k}\right)\zeta \\ {\tilde{z}}_{B}=\left({\tilde{z}}_{p,k}/{\tilde{x}}_{p,k}\right)\zeta \end{array}.$$

Else if ${\tilde{x}}_{p,k}<0$, then

(17) $$\{\begin{array}{l} {\tilde{x}}_{A}=\zeta \\ {\tilde{y}}_{A}=\left({\tilde{y}}_{p,k}/{\tilde{x}}_{p,k}\right)\zeta \\ {\tilde{z}}_{A}=\left({\tilde{z}}_{p,k}/{\tilde{x}}_{p,k}\right)\zeta \end{array},\;\{\begin{array}{l} {\tilde{x}}_{B}=-\zeta \\ {\tilde{y}}_{B}=-\left({\tilde{y}}_{p,k}/{\tilde{x}}_{p,k}\right)\zeta \\ {\tilde{z}}_{B}=-\left({\tilde{z}}_{p,k}/{\tilde{x}}_{p,k}\right)\zeta \end{array}.$$

in which,

(18) $$\zeta ={\left(\frac{1}{a^{2}}+\frac{1}{b^{2}}{\left(\frac{{\tilde{y}}_{p,k}}{{\tilde{x}}_{p,k}}\right)}^{2}+\frac{1}{c^{2}}{\left(\frac{{\tilde{z}}_{p,k}}{{\tilde{x}}_{p,k}}\right)}^{2}\right)}^{-1/2}.$$

Define the circular sections passing through points *A* and *B*, and perpendicular to the cone axis as $S_{A}$ and $S_{B}$, respectively. Subsequently, their radii are

(19) $$r_{A}=tg\theta_{G}\sqrt{{\left({\tilde{x}}_{A}-{\tilde{x}}_{p,k}\right)}^{2}+{\left({\tilde{y}}_{A}-{\tilde{y}}_{p,k}\right)}^{2}+{\left({\tilde{z}}_{A}-{\tilde{z}}_{p,k}\right)}^{2}}.$$

(20) $$r_{B}=tg\theta_{G}\sqrt{{\left({\tilde{x}}_{B}-{\tilde{x}}_{p,k}\right)}^{2}+{\left({\tilde{y}}_{B}-{\tilde{y}}_{p,k}\right)}^{2}+{\left({\tilde{z}}_{B}-{\tilde{z}}_{p,k}\right)}^{2}}.$$

If the point *P* lies outside the ellipsoid, which means

(21) $$\frac{{\tilde{x}}_{p,k}^{2}}{a^{2}}+\frac{{\tilde{y}}_{p,k}^{2}}{b^{2}}+\frac{{\tilde{z}}_{p,k}^{2}}{c^{2}}>1,$$

Subsequently, the volume of the MDC gate can be approximated by the volume of the truncated cone between $S_{A}$ and $S_{B}$, which is

(22) $$V_{\theta -G}\approx V_{b}=\frac{1}{3}\pi h(r_{A}^{2}+r_{B}^{2}+r_{A}r_{B}).$$

where the height of the cone is $h=\sqrt{{\left({\tilde{x}}_{A}-{\tilde{x}}_{B}\right)}^{2}+{\left({\tilde{y}}_{A}-{\tilde{y}}_{B}\right)}^{2}+{\left({\tilde{z}}_{A}-{\tilde{z}}_{B}\right)}^{2}}$.

If the point *P* is inside the ellipsoid, then

(23) $$\frac{{\tilde{x}}_{p,k}^{2}}{a^{2}}+\frac{{\tilde{y}}_{p,k}^{2}}{b^{2}}+\frac{{\tilde{z}}_{p,k}^{2}}{c^{2}}\leq 1.$$

In this case, the volume of the MDC gate can be approximated by the volume of the cone between $S_{A}$ and point *P*, which is

(24) $$V_{\theta -G}\approx \frac{1}{3}\pi r_{A}^{2}\sqrt{{\left({\tilde{x}}_{A}-{\tilde{x}}_{p,k}\right)}^{2}+{\left({\tilde{y}}_{A}-{\tilde{y}}_{p,k}\right)}^{2}+{\left({\tilde{z}}_{A}-{\tilde{z}}_{p,k}\right)}^{2}}.$$

The expressions given in Equations (22) and (24) provide very effective approximation when the volume of the MDC gate is notably smaller than that of the conventional ellipsoidal gate. However, when the innovation covariance is small, the volume of the ellipsoidal gate can be comparable or even smaller than the MDC gate, where, in the latter case, the ellipsoidal gate is located inside the cone. Thus, a simple rule is adopted: if the point *P* lies outside of the ellipsoidal gate and satisfies $V_{G}\leq \kappa \cdot V_{b}$, κ≥1, then we set $V_{\theta -G}=V_{G}$, in which κ is a constant coefficient and it can be obtained by the simulation. In real target tracking applications, the coefficient κ represents the certainty degree of the prior target maneuvering information, and its value affects the effect of the motion direction constraint. When the value of κ is big, the influence of the motion direction constraint will reduce and vice versa. In this paper, we set κ=1.

## 4. MHT Using Validation Gate with Motion Direction Constraint

**Figure 2:**
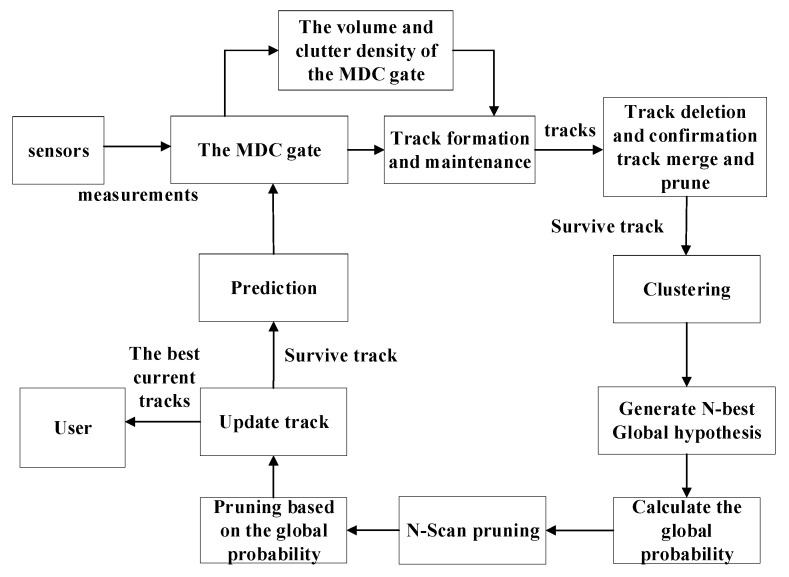
The flowchart of MHT-MDC.

In this section, a new multiple hypothesis tracker using validation gate with motion direction constraint (MHT-MDC) is proposed. The MHT-MDC flowchart that is based on the TOMHT logic [14,15,16] is shown in Figure 2. We utilize the prior target maneuvering information to design the MDC gate, and then calculate the gate volume and estimate the clutter density in the gate. For the existing tracks, the track-to-measurement association hypotheses are formed at the current scan, and their score functions are computed using the estimated clutter density. Subsequently, the track deletion and confirmation are carried out based on the track scores. Subsequently, we implement the clustering process in surviving tracks and calculate the global hypothesis of each cluster. The track pruning step is then conducted based on the global hypothesis. Finally, the surviving tracks are updated for the next scan. The MHT-MDC aims to reduce the uncertainty of track-to-measurement association hypothesis by using the MDC gate, and improve the quality of the score function by applying the CMECD to adaptively estimate the clutter density. When compared to the traditional MHT, the MHT-MDC can effectively improve the tracking performance for weak maneuvering targets in heterogeneous and dense clutter scenarios.

At scan *k*, we assume that there are *N* tracks $T_{t}(k-1),t=1,2,\cdots ,N$. For track *t*, its innovation covariance is expressed as $S_{t}(k)$. Based on Section 3, the volume of the MDC gate can be calculated as $V_{\theta -G}^{t}$, and the number of validation measurements in the MDC gate is $M_{k}^{t}$.

### 4.1. The Clutter Density Estimation

In the tracking process, the spatial distribution of the clutter is usually assumed to be a Poisson distribution [17,18], and the probability mass function (PMF) of the clutter within the validation gate can be defined as

(25) $$P(l)={\left(\frac{\lambda V}{l!}\right)}^{l}e^{-\lambda V}.$$

Here, l is the false alarms generated by the clutter in the gate, λ is clutter density, and V is the volume of the gate. In practical tracking applications, if the clutter has a small density and obeys a uniform distribution, then the clutter density λ can be set as a constant. However, in a scene with dense and heterogeneous clutter, the setting of a constant clutter density will lead to the increase in the number of false alarm tracks and fragmentary tracks, which can severely deteriorate the tracking performance. It is necessary to adaptively estimate the clutter density at different positions to solve these practical problems.

The conditional mean estimator of clutter density (CMECD) [19,20] is utilized to improve the accuracy of clutter density estimation. According to the references [19,20], the mean number of selected clutter in MDC gate with volume $V_{\theta -G}^{t}$ can be estimated as

(26) $${\hat{M}}_{k}^{t}=M_{k}^{t}-\frac{\tau M_{k}^{t}/V_{\theta -G}^{t}}{{\hat{\lambda }}_{c}^{t}(k)+\tau M_{k}^{t}/V_{\theta -G}^{t}}.$$

where $M_{k}^{t}$ is the measurement number in the MDC gate, ${\hat{\lambda }}_{c}^{t}(k)$ is the estimated clutter density in the MCD gate at scan *k*. The parameter τ is defined as

(27) $$\tau =\frac{P_{D}P_{G}\bar{\psi }}{1-P_{D}P_{G}\bar{\psi }}.$$

where $P_{D}$ is the target detection probability and $\bar{\psi }$ is the existence probability of predicted target.

As ${\hat{M}}_{k}^{t}={\hat{\lambda }}_{c}^{t}(k)V_{\theta -G}^{t}$, based on the maximum likelihood estimation principle, the clutter density in the MDC gate can be calculated as

(28) $${\hat{\lambda }}_{c}^{t}(k)=\frac{M_{k}^{t}}{2V_{\theta -G}^{t}}\left(1-\tau +\sqrt{{\left(1-\tau \right)}^{2}+4\tau (M_{k}^{t}-1)/M_{k}^{t}}\right).$$

### 4.2. The Track Score

In the MHT algorithm, each track hypothesis has a corresponding track score, which is a log-likelihood ratio (LLR) of the association hypothesis. In the traditional definition of the score function, the clutter density is set as a constant. In scenarios with dense and heterogeneous clutter, the error of track score will grow, which will lower the accuracy of data association. In this section, the clutter density in the MDC gate is utilized in order to calculate the score function to improve the data association accuracy.

At scan *k*, the track score can be calculated recursively as

(29) $$L\left\{T_{t}(k)\right\}=L\left\{T_{t}(k-1)\right\}+\Delta L_{m}^{t}(k).$$

We assume that there exist $M_{k}^{t}$ measurements $z_{m}(k),m=1,2,\cdots ,M_{k}^{t}$ in the validation gate of track $T_{t}(k-1)$. $z_{0}$ represents the absence of measurement. Subsequently, the increment term of the corresponding track score can be calculated as

(30) $$\Delta L_{m}^{t}(k)=\left\{ \begin{array}{cc} \ln(1-P_{D}), & m=0\\ \ln\left[\frac{p\left\{z_{m}(k)|T_{t}(k-1)\right\}P_{D}}{\lambda_{v}+\lambda_{c}}\right], & m\neq 0 \end{array}\right..$$

where $\lambda_{v},\lambda_{c}$ denote the density of the new target and the clutter, respectively. The detection probability is $P_{D}$. If there is no association hypothesis at scan *k*, the score increment will be $\ln(1-P_{d})$. In Equation (30), when the track $T_{t}(k-1)$ exists at scan *k* − 1, the likelihood of the measurement $z_{m}(k)$ is

(31) $$p\left\{z_{m}(k)|T_{t}(k-1)\right\}=\frac{1}{{\left(2\pi \right)}^{3/2}{\left|S_{t}(k)\right|}^{1/2}}\exp\left\{-\frac{1}{2}v_{m}{}^{T}S_{t}(k)v_{m}\right\}.$$

In MHT-MDC, the score increment for measurement $z_{m}(k)$ in MDC gate can be represented as

(32) $$\Delta L_{m}^{t}(k)=\left\{ \begin{array}{ll} \ln(1-P_{D}), & m=0\\ \ln\left[\frac{p\left\{z_{m}(k)|T_{t}(k-1)\right\}P_{D}}{\lambda_{v}+{\hat{\lambda }}_{c}^{t}(k)}\right], & m\neq 0 \end{array}\right..$$

where ${\hat{\lambda }}_{c}^{t}(k)$ is the clutter density that is adaptively estimated in the MDC gate by Equation (28).

The sequential probability ratio test (SPRT) is used to determine the status of the track by comparing its current score in Equation (29) with both the lower threshold and the upper threshold [21]. Generally, the track score over the upper threshold will be confirmed, and the track score below the lower threshold will be deleted; other tracks will remain for the further test. The SPRT can be defined as

(33) $$L\{T_{t}(k)\}\left\{ \begin{array}{cc} \leq \ln(\left(1-\beta \right)/\alpha ), & delete\\ \geq \ln(\beta /\left(1-\alpha \right)), & comfirm\\ otherwie, & continue\;test \end{array}\right..$$

where α is the false track confirmation probability and β is the true track deletion probability.

### 4.3. The Global Hypothesis Generation

The clustering technique reduces the combinatorial complexity by decomposing the whole tracks into clusters. A cluster is formed by a collection of incompatible tracks, each cluster can generate its global hypotheses and independently calculate its global probability of trajectory. In TOMHT, the commonly used algorithms to search for the global optimal hypothesis include the Lagrangian relaxation implementation algorithm, the multiple dimensional assignment (MDA) algorithm, and the greedy randomized adaptive search procedure (GRASP) method [22,23]. Assume that there are *J* global hypotheses. Thus, the *i*th global hypothesis $H_{i}(k)$ at scan *k* can be calculated as

(34) $$L\left\{H_{i}(k)\right\}=\sum_{T_{j}(k)\in H_{i}(k)}L\left\{T_{j}(k)\right\}.$$

The probability of the global hypothesis $H_{i}(k)$ can be expressed as

(35) $$P\left\{H_{i}(k)\right\}=\frac{\exp\left(L\left\{H_{i}(k)\right\}\right)}{1+\sum_{j=1}^{J}\exp\left(L\left\{H_{i}(k)\right\}\right)}.$$

Thus, the global probability of track $T_{j}(k)$ can be denoted as

(36) $$P\left\{T_{j}(k)\right\}=\sum_{T_{j}(k)\in H_{i}(k)}P\left\{H_{i}(k)\right\}.$$

### 4.4. Pruning

*N*-scan pruning strategy is another essential technique to reduce the computational burden by limiting the depth of the track tree. After obtaining the global optimal hypothesis, the *N*-scan pruning strategy retains all tracks that share a common root with the global optimal hypothesis when the depth of the track tree more than *N*, and the others will be deleted.

Further, the surviving tracks will be pruned by evaluating its posterior probability of the hypothesis track, and the final surviving tracks will be delivered to the next scan.

## 5. Experimental Results

In this section, the experimental results of two simulation scenarios are given. When compared with the MHT, the tracking performance of the MHT-MDC is verified by evaluating a set of performance metrics.

### 5.1. Simulation Scenario

The following two different challenging scenarios are introduced.

**Figure 3:**
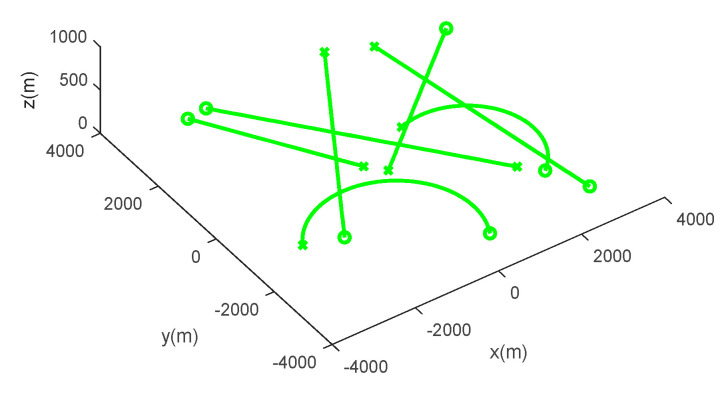
Target trajectories in scenario 1.

**Figure 4:**
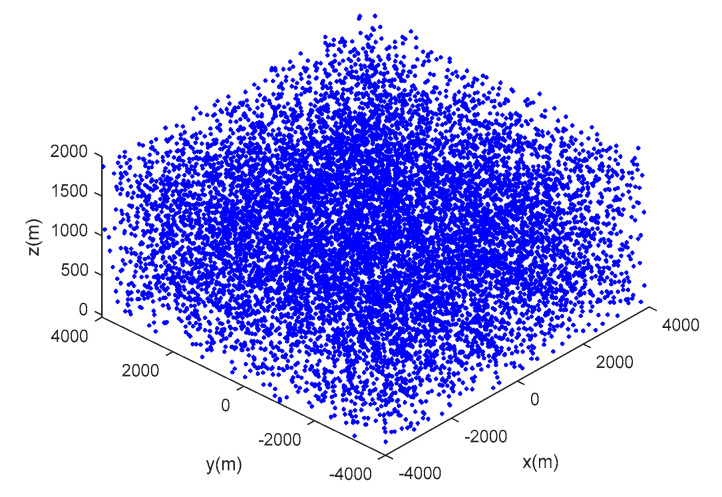
The real measurements with clutter in scenario 1.

Scenario 1: seven moving targets with intersecting tracks are distributed in a space of [−4000, 4000 m] × [−4000, 4000 m] × [0, 1000 m], and the true movement of targets is shown in Table 1. Figure 3 shows the target trajectories in scenario 1, and the start and end points of tracks are tagged as ∘,×, respectively. Figure 4 shows the measurements with dense clutter in scenario 1.

**Figure 5:**
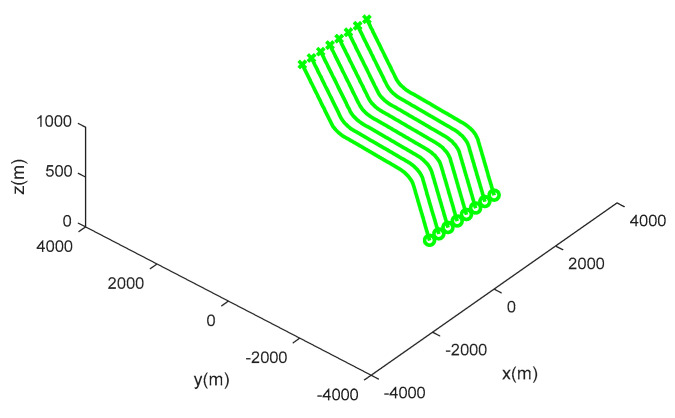
Target trajectories in scenario 2.

**Figure 6:**
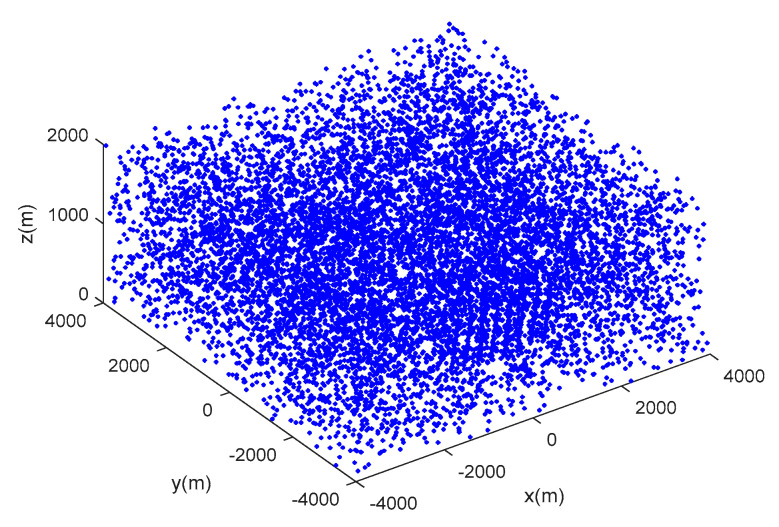
The real measurements with clutter in scenario 2.

Scenario 2: here, we consider eight moving targets with the paralleling tracks, and the targets are moving in formation with a separation of 300 m and a speed of 71 m/s over 100 s. Each target takes two turns in the 30 s and 60 s, and each turn lasts for 10 s. Figure 5 shows the trajectories of targets in scenario 2, and Figure 6 shows the target measurements with dense clutter in scenario 2.

For all of these scenarios, the detection probability $P_{D}=0.95$, and the average clutter number value in different regions are randomly generated within the range of $\lambda =1\times 10^{-4}/m^{3}\sim 3\times 10^{-4}/m^{3}$. The standard deviation of process noise $\delta_{v}=6m/s^{2}$ and the standard deviation of the measurement noise $\delta_{c}=2m$. For SPRT, the true track deletion probability $\beta =10^{-3}$, and the false track confirmation probability $\alpha =10^{-6}$. The new target density $\lambda_{v}=1\times 10^{-13}/m^{3}$. The sample interval T=1 s, the number of scans is set to 100, the motion direction threshold $\theta_{G}=\pi /3$, and the depth of TOMHT is set to 6. All of the performance metrics obtained by these algorithms are evaluated over 100 Monte-Carlo simulations.

### 5.2. Results and Evaluation

In this part, the performance evaluation of two algorithms is obtained by following widely used metrics [24,25,26,27].

(1) The fragmentary ratio of true tracks $R_{FT}$. The ratio of the true track fragmentary in the tracking results, that is the ratio of the number of true track fragmentary times to the truth track number.
(2) The miscorrelation ratio of true tracks $R_{MC}$. If the track is associated with the measurement in which origination differs from the track, it can be considered as the miscorrelation measurement. $R_{MC}$ is defined as the ratio of the total number of miscorrelation measurements over the sum of true track life, which describes the data association quality.
(3) The correct correlation ratio of true tracks $R_{CC}$. $R_{CC}$ is defined as the ratio of the number of correct measurement-to-track pairings to the number of the observations that originate from the target, which describes the tracking accuracy rate of the true tracks.
(4) The average processing time per scan $T_{H}$. The execution time for one run, which can evaluate the algorithm computational complexity in seconds.
(4) The optimal sub-pattern assignment (OSPA) distance. The OSPA distance can evaluate the cardinality and position estimation error for multi-target tracking. The set of the real position and the estimated position of the target can be denoted as $X=\left\{x_{1},x_{2},\cdots ,x_{m}\right\}$ and $Y=\left\{y_{1},y_{2},\cdots ,y_{m}\right\}$, respectively. The OSPA distance can be defined as

(37) $$d_{p}^{\left(c\right)}(X,Y)={\left\{\frac{1}{n}\left(\underset{\pi \in \Pi_{n}}{\min}\sum_{i=1}^{m}d^{\left(c\right)}{\left(x_{i},y_{\pi (i)}\right)}^{p}+c^{p}(n-1)\right)\right\}}^{\frac{1}{p}},n>m.$$

when n≤m, $d_{p}^{\left(c\right)}(X,Y)≜d_{p}^{\left(c\right)}(Y,X)$. Define $\Pi_{n}$ as the set of all possible permutations of {1,2,⋯,n}, and $d^{\left(c\right)}(X,Y)≜\min\left(c,\left\|x-y\right\|\right)$ represents the truncated Euclidean distance between x and y, ‖·‖ denotes the Euclidean distance. In all simulations, the cut-off parameter and the order parameter are set as c=100 and p=1, respectively.

**Figure 7:**
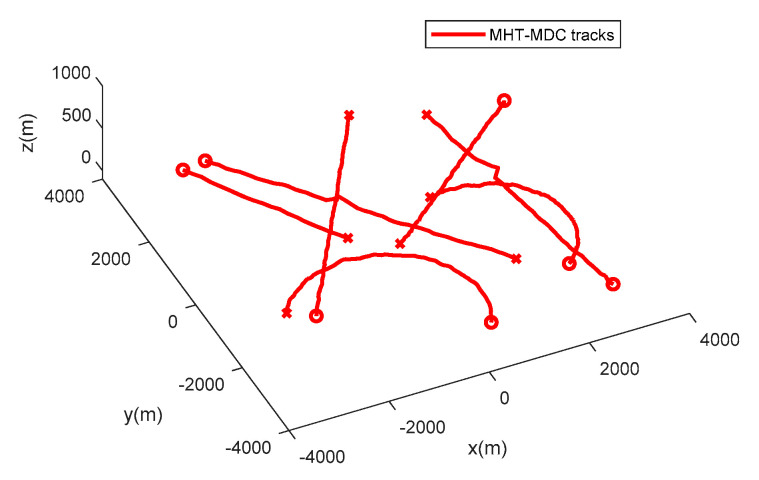
The estimated trajectories of the multiple hypothesis tracker using validation gate with motion direction constraint (MHT-MDC) in scenario 1.

**Figure 8:**
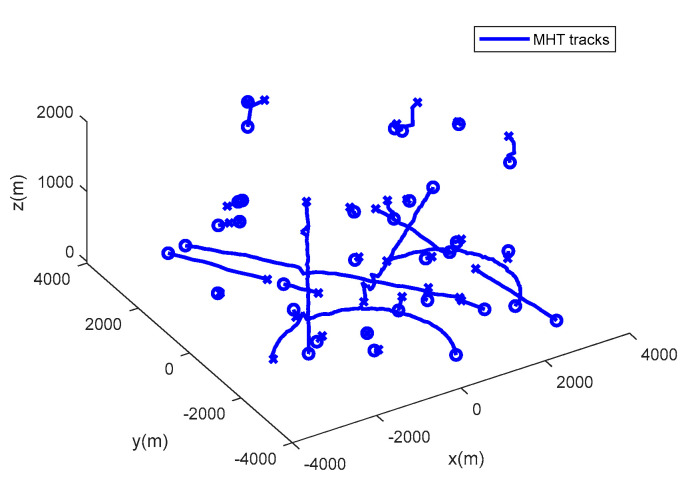
The estimated trajectories of the MHT in scenario 1.

**Figure 9:**
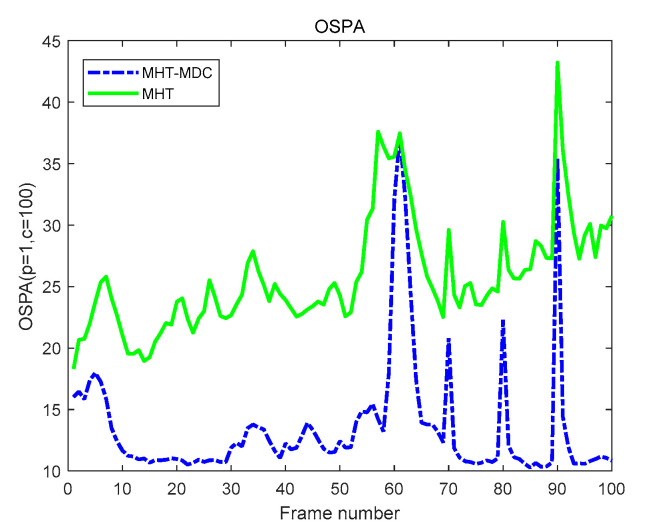
The optimal sub-pattern assignment (OSPA) distance of two algorithms in scenario 1.

**Figure 10:**
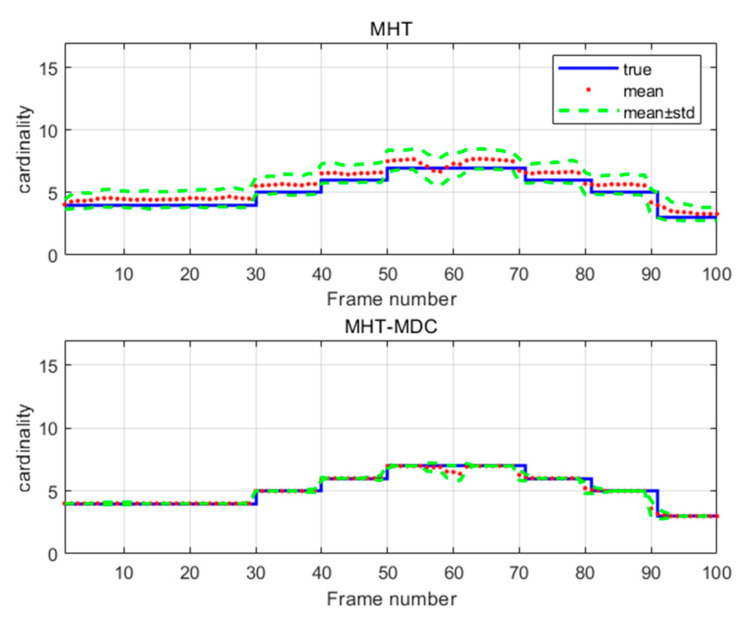
The cardinality estimation of two algorithms in scenario 1.

The performance of the MHT-MDC and the MHT in scenario 1 are presented in Table 2 and Figure 7, Figure 8, Figure 9 and Figure 10. In Figure 7 and Figure 8, it is clear that the MHT-MDC obtains better tracking quality when compared with the MHT, as it has fewer false tracks and less mutual interference. Figure 9 and Figure 10 show the average OSPA distance and the cardinality estimation over 100 Monte Carlo runs. When clutter is dense, the MHT suffers inaccuracy cardinality estimation, which leads to a higher average OSPA distance and poorer tracking accuracy. In contrast, the MHT-MDC has a lower average OSPA distance and more accurate cardinality estimation.

According to Table 2, we can figure out that the MHT-MDC has a higher correct correlation rate $R_{CC}$ and a lower miscorrelation rate $R_{MC}$, which demonstrates that the MHT-MDC has better data association accuracy. We can see that it has a smaller fragmentary ratio of $R_{FT}$ and a lower average processing time $T_{H}$, which shows that the MHT-MDC also acquires better track quality and lower computational complexity.

**Figure 11:**
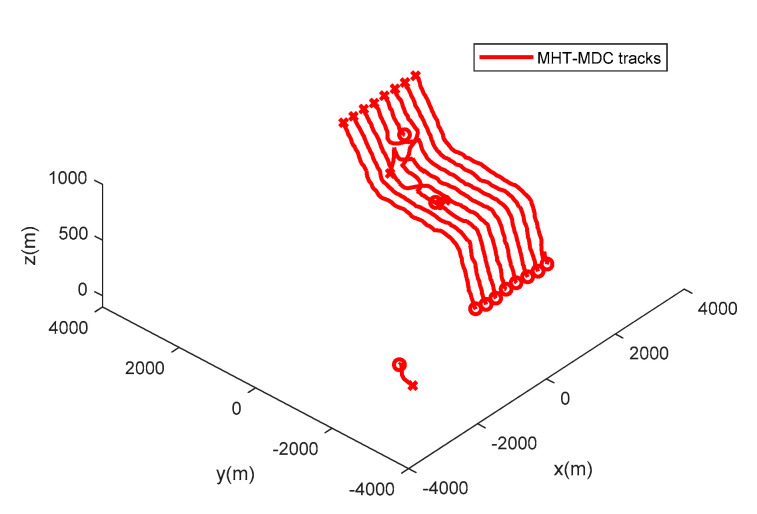
The estimated trajectories of the MHT-MDC in scenario 2.

**Figure 12:**
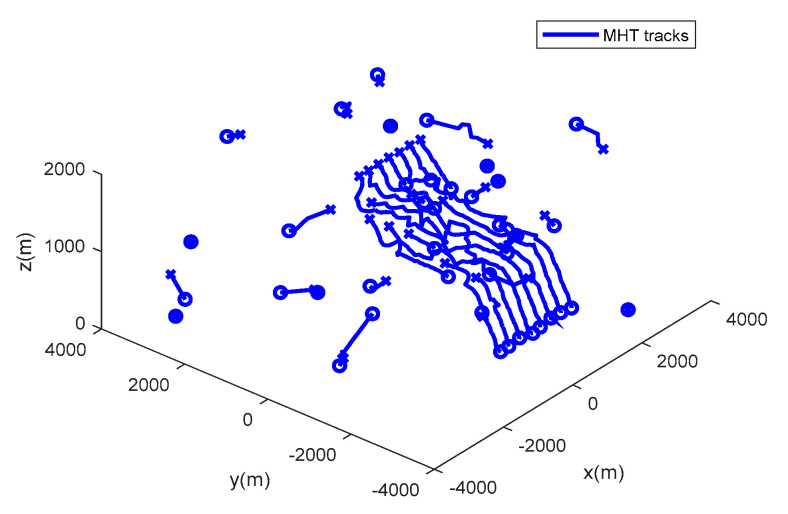
The estimated trajectories of the MHT in scenario 2.

**Figure 13:**
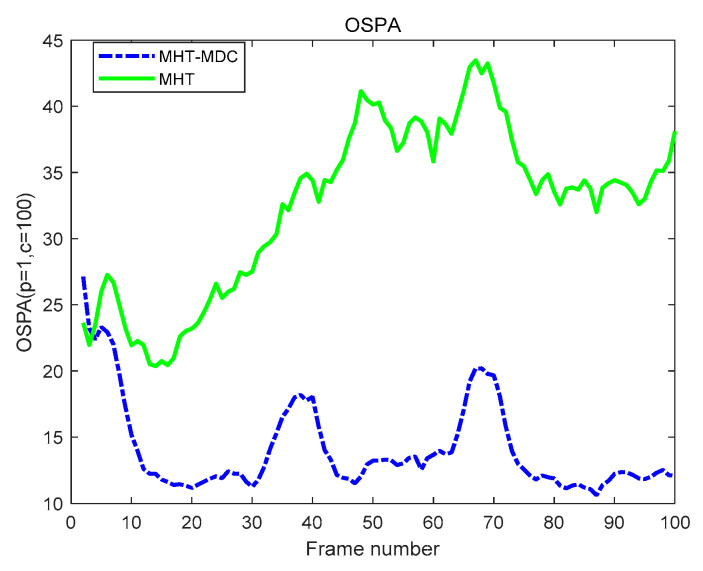
The OSPA distance of two algorithms in scenario 2.

**Figure 14:**
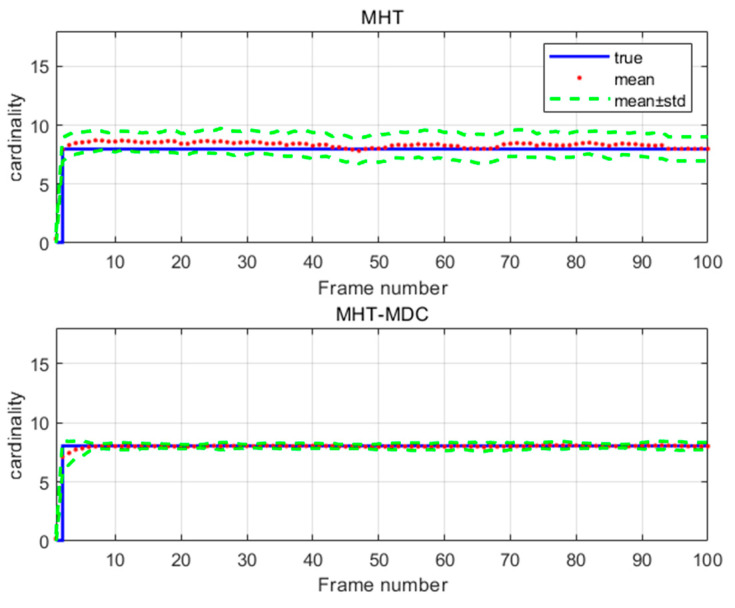
The cardinality estimation of two algorithms in scenario 2.

The performance of the MHT-MDC and the MHT in scenario 2 are presented in Table 3 and Figure 11, Figure 12, Figure 13 and Figure 14. Figure 11 and Figure 12 show the estimated tracks from the MHT and the MHT-MDC in scenario 2, respectively. We can observe that the MHT-MDC outputs cleaner tracks and also effectively decreased the number of false tracks and, thus, we can conclude the MHT-MDC acquires better tracking performance. In Figure 13 and Figure 14, the MHT-MDC has a lower average OSPA distance, a smaller cardinality estimation covariance, and a more accurate cardinality estimation, which illustrates that it can acquire better estimation performance.

Table 3 displays the tracking performance of the MHT-MDC and the MHT in the association quality and average processing time. We can conclude that the MHT-MDC achieves higher track continuity and data association accuracy, as it has a higher $R_{CC}$, a lower $R_{MC}$, and a smaller $R_{FT}$. The MHT-MDC also proves to be more efficient, as it has a comparatively lower average processing time.

## 6. Conclusions

In this letter, we propose a new multiple hypothesis tracker using validation gate with motion direction constraint (MHT-MDC), which can effectively improve the multi-target tracking performance in the scenarios with dense and heterogeneous clutter. The simulation results show that the MHT-MDC can successfully reduce the number of false measurements within the validation gate and, thus, can restrain the false alarm tracks and improve the speed of computation. In addition, the MHT-MDC outperforms the MHT in data association with a higher correct correlation ratio of true tracks and a lower fragmentary ratio of true tracks. In our simulation scenarios, the average OSPA distances of the MHT-MDC are smaller than half of the standard MHT’s. There is room for future development of the MHT-MDC: for example, combining the other *a* prior information aided tracking algorithm; using the *a* prior information from the estimated target trajectory (e.g., the continuous-time trajectory information [28], the estimated target velocity and the location information) in order to improve the tracking performance.

## Figures and Tables

**Table 1:** The true movement of targets.

Initial State of Target	Life Time
[500 m, 30 m/s, −3000 m, 50 m/s, 0 m, 0 m/s]	(1~100 s)
[2500 m, 40 m/s, −2000 m, 30 m/s, 700 m, 0 m/s]	(1~90 s)
[−3000 m, 30 m/s, −3000 m, 50 m/s, 0 m, 0 m/s]	(1~100 s)
[3500 m, −35 m/s, 2800 m, −30 m/s, 0 m, 0 m/s]	(1~100 s)
[−3000 m, 80 m/s, 2000 m, −100 m/s, 700 m, 0 m/s]	(40~90 s)
[3000 m, −20 m/s, −3000 m, 120 m/s, 0 m, 0 m/s]	(30~80 s)
[−3500 m, 100 m/s, 2000 m, −160 m/s, 700 m, 0 m/s]	(50~70 s)

**Table 2:** The performance metrics of two algorithms in scenario 1.

Algorithm	$R_{FT}$	$R_{MC}$	$R_{CC}$	$T_{H}$
MHT	0.357	0.074	0.917	0.134 s
MHT-MDC	0.11	0.017	0.972	0.084 s

**Table 3:** The performance metrics of two algorithms in scenario 2.

Algorithm	$R_{FT}$	$R_{MC}$	$R_{CC}$	$T_{H}$
MHT	0.731	0.206	0.612	0.258 s
MHT-MDC	0.086	0.049	0.93	0.195 s
